# Identifying and prioritizing key risk factors in internet-based shared nursing services from the nurses’ perspective: a hybrid multi-criteria decision-making approach

**DOI:** 10.3389/fpubh.2026.1956352

**Published:** 2026-10-19

**Authors:** Jiechenming Xiao, Heng Yang, Weihong Zhang, Lihua Wang, Dan Xu

**Affiliations:** Department of Nursing, Taizhou First People’s Hospital, Taizhou, Zhejiang, China

**Keywords:** consistent fuzzy preference relations, decision making, importance–performance analysis, internet plus, shared nursing services

## Abstract

**Introduction:**

Home visiting programs are constrained by nursing shortages and limited coverage. Internet-based shared nursing services in China offer a potential solution but introduce multidimensional risks that have not been systematically examined from the perspective of shared nurses, the primary stakeholders in service safety. This study aimed to identify and prioritize key risk factors from the nurses’ perspective to facilitate targeted risk management strategies.

**Methods:**

A purposive sample of 30 shared nurses from a tertiary general hospital in Zhejiang Province, China, completed a survey in March 2026 based on the Internet-based Risk Factor (IBRF) Scale for Shared Nursing Services. Within a hybrid multi-criteria decision-making model, consistent fuzzy preference relations (CFPR) were used to assign weights to the risk factors, and importance–performance analysis (IPA) was applied to identify management gaps and prioritize interventions.

**Results:**

At the dimension level, nurses’ clinical core competence (C5), patient/family risk assessment (C1), and nurses’ service-related experience (C4) had the highest weights. At the item level, the top five risk factors were the establishment of trust with patients/families (C13), the fulfillment of expected service outcomes (C14), the adequacy of systematic home-care training (C43), proficiency in clinical problem-solving and emergency handling (C54), and the ability to remain calm in difficult situations (C55). IPA identified patient/family courtesy (C11), educational background (C12), and the establishment of trust (C13) as high-importance–low-performance priorities.

**Discussion:**

Based on data from a single tertiary hospital, this study provides a systematic framework for prioritizing risk factors in shared nursing services. Concrete strategies—such as communication training modules for nurses, platform-based risk alert systems, age-tailored public education, and nurse protection mechanisms— may reduce nurses’ perceived risks; however, multicenter validation is warranted.

## 1 Introduction

With the growing demand for diverse medical services, community health services have expanded substantially, becoming an indispensable component of the healthcare system. Home visiting, a core component of community nursing, involves healthcare professionals providing professional nursing or medical services within patients’ homes to promote and maintain health (1). Home visiting programs have been implemented in several countries, including the United Kingdom (2), Germany (3), Japan (4), and China (5), demonstrating positive effects on health promotion and disease management. For example, home visits have been shown to improve the quality of life for patients with asthma, to support blood glucose and blood pressure management among patients with diabetes and those with hypertension, and to substantially reduce healthcare expenditures among older adults with disabilities (6–8).

Despite these benefits, promoting and implementing home visiting services face substantial challenges; notably, the shortage of nursing personnel is a major barrier to service delivery (9). In China, home visiting services are primarily community-based, delivered by registered community nurses. Although this model provides a broad range of care services, shortages of primary healthcare personnel and geographically restricted service coverage limit the ability of community nurses to meet the growing demand for home-based care (10).

The rapid development of Internet technologies has provided a potential solution to these challenges. In China, shared nursing services—officially promoted as “Internet Plus Nursing Services” and delivered through online nurse-booking platforms—have emerged as an innovative service model. Through these platforms, qualified registered nurses from healthcare institutions can register to provide home-based nursing services as “shared nurses” (11). Since 2019, the National Health Commission of the People’s Republic of China has issued policy documents encouraging innovative nursing service models and launched pilot programs for shared nursing services in six provincial-level regions (Beijing, Tianjin, Shanghai, Jiangsu, Zhejiang, and Guangdong) and selected cities in other provinces; the model has since been rapidly scaled up nationwide (12). By integrating nursing personnel from healthcare institutions into digital platforms, this model may improve the efficiency of matching available resources with patient needs. However, shifting nursing care from institutional settings to patients’ homes via digital platforms may simultaneously introduce new risks that differ fundamentally from those in hospital-based care.

These risks are multidimensional and have not been systematically identified or evaluated. International studies have documented various occupational exposures faced by home healthcare workers within patients’ homes, client-perpetrated violence during home visits, and broader safety concerns in home care nursing; risks related to data privacy and information security on digital platforms have also been reported (13–16). In the Chinese context, surveys indicate that nurses’ willingness to participate in Internet-based nursing services is closely associated with their perceptions of occupational risk and the availability of safety protections (17, 18). However, existing research on shared nursing services has primarily focused on service effectiveness (19), residents’ willingness to use such services (20), and nurses’ willingness to provide them (21, 22); risk-related studies have mainly described nurses’ risk perception levels and their associated determinants or qualitatively explored service experiences (23, 24). Studies that systematically identify the key risk factors, rank their relative importance, and link them to actionable management priorities remain lacking.

Because shared nurses are the direct providers of these services, their risk perceptions and decision-making behaviors are critical determinants of risk generation and prevention; therefore, understanding the risks they perceive is essential for the safe and sustainable development of this service model. To systematically identify and prioritize these risks, this study applied a hybrid multi-criteria decision-making model combining consistent fuzzy preference relations (CFPR) and importance–performance analysis (IPA), using the Internet-based Risk Factor (IBRF) scale as the risk-factor framework (25). Specifically, this study aimed to identify and assign weights to the key risk factors associated with shared nursing services from the perspective of shared nurses, map these factors against current management performance to derive improvement priorities, and propose targeted risk management strategies accordingly. The findings are expected to inform resource allocation by healthcare institutions and platform operators and to support policy development for the safe, sustainable growth of shared nursing services.

## 2 Methods

### 2.1 Study design and theoretical framework

This study aimed to identify and prioritize key risk factors associated with Internet-based shared nursing services from the perspective of shared nurses and to propose targeted risk management strategies. A hybrid multi-criteria decision-making (MCDM) model integrating CFPR and IPA was adopted. The IBRF scale provided the validated risk-factor framework. CFPR was used to derive the relative weights of risk dimensions and items based on pairwise comparisons made by an expert panel of shared nurses. IPA was then applied to map these weights against current management performance, thereby identifying high-priority improvement areas. Shared nurses were selected as the expert panel because, as they are the direct providers of home-based services, their risk perceptions and decision-making behaviors are critical determinants of risk generation and prevention.

### 2.2 Participants and sample size

Participants were recruited from a tertiary general hospital in Zhejiang Province, one of the first pilot regions for shared nursing services in China. The inclusion criteria were as follows: (1) registration on the shared nurse platform, (2) qualification to provide home-based nursing services, (3) participation in shared nursing services for at least 6 months, and (4) voluntary participation with informed consent. Nurses who had not participated in shared nursing services within the past 3 months were excluded. No formal statistical power calculation was performed because this study adopted an MCDM approach rather than a hypothesis-testing design. CFPR requires only n − 1 pairwise comparisons per criterion level and relies on the consistency of expert judgments rather than on sample size for statistical representativeness. In previous MCDM studies using CFPR or related pairwise comparison techniques, expert panel sizes have typically ranged from 15 to 30 (26–28). The 30 shared nurses included in this study fell within this recommended range, and all possessed at least 6 months of frontline experience in shared nursing services.

### 2.3 Instrument: the IBRF scale

The IBRF scale was adapted from the “Risk Factors” section of the “Nurse Risk Factor and Risk Outcome Assessment Scale for Internet Plus Nursing Services for Older adults” developed by Xu et al. (25). The original instrument contains two sections assessing risk factors and risk outcomes, respectively. Because the present study focused specifically on nurses’ perceptions of risk factors during service delivery, only the “Risk Factors” section was used. The IBRF scale consists of five dimensions and 27 items. It employs a 5-point Likert format to measure perceived risk levels. The original scale demonstrated excellent psychometric properties, including an overall Cronbach’s α of 0.970, dimension-specific Cronbach’s α values of 0.803–0.973, a content validity index of 0.941, and a cumulative variance contribution of 84.504% (25). In this study, the IBRF scale served as the content-validated framework and item pool for identifying risk dimensions and items. For brevity, the five dimensions and 27 items are coded as C1–C5 and C11–C55, respectively, as listed in Table 1. Participants also rated the importance of each item on a 5-point Likert scale (1 = very unimportant, 5 = very important) to facilitate preliminary ranking. However, these 5-point importance ratings were not used to calculate the CFPR weights.

**Table 1:** The Internet-based Risk Factor (IBRF) scale for shared nursing services.

Dimension	Item
C1: Patient/Family risk assessment	C11: Patient/family courtesy
	C12: Patient/family educational background
	C13: Establishment of a trust relationship with patients/family members
	C14: Consistency of service outcomes with the expectations of patients/family members
C2: External environment risk assessment	C21: Favorability of weather on the service day
	C22: Appropriateness of the distance between the service location and the departure point
	C23: Safety of transportation on the service day
	C24: Condition of roads and convenience of transportation on the service day
	C25: Accessibility of the residential community and convenience of surrounding transportation
	C26: Security and safety of the residential community environment
	C27: Safety of the client’s home environment (e.g., unobstructed passages, non-slip floors)
	C28: Adequacy of lighting in the client’s home and nursing operation environment
C3: Platform/Hospital supply risk assessment	C31: Provision of effective supplies within their shelf life by the platform/hospital
	C32: Provision of work recorders by the platform/hospital to ensure service transparency
	C33: Provision of app-based location tracking and one-click alarm devices by the platform/hospital
	C34: Information and data security of the platform/hospital
	C35: Reasonableness of service pricing by the platform/hospital
	C36: Provision of medical liability and personal accident insurance for nurses by the platform/hospital
C4: Nurses’ service-related experience	C41: Institutional encouragement of Internet Plus Nursing Services home visits
	C42: Understanding of qualification requirements and relevant laws and regulations
	C43: Adequacy of systematic and comprehensive home-care training
	C44: Level of experience in Internet Plus Nursing Services (≥3 participations)
C5: Nurses’ clinical core competence assessment	C51: Alignment of professional knowledge with service requirements
	C52: Proficiency in nursing operation skills
	C53: Effectiveness of communication with patients and their families
	C54: Proficiency in accurately assessing clinical problems and performing emergency handling
	C55: Ability to remain calm and orderly when dealing with difficult clinical problems

### 2.4 CFPR procedure

Proposed by Herrera-Viedma et al. (29), the CFPR method constructs a logically consistent fuzzy preference matrix based on additive consistency. For each set of criteria at the same hierarchical level, experts perform only *n* − 1 pairwise comparisons, substantially reducing respondent burden compared with the traditional analytic hierarchy process (AHP), which requires *n*(*n* − 1)/2 comparisons (29, 30). In this study, the 30 shared nurses acted as the expert panel. At the dimension level, each expert completed four pairwise comparisons among the five dimensions. At the item level, each expert completed *n* − 1 pairwise comparisons within each dimension. Before each pairwise comparison task, participants ranked the relative importance of the dimensions or items. They then compared each pair using a 1–9 linguistic scale. These linguistic judgments were transformed into fuzzy preference values, and the CFPR algorithm based on additive consistency was applied to construct complete fuzzy preference relations and to derive local and global weights. Consistency analysis was performed to verify the logical consistency of the expert judgments. Owing to these advantages, the CFPR method has been widely applied in various fields, including the prediction of knowledge implementation success (31), supplier selection (32), and the evaluation of shared decision-making quality based on orthopedic nurses’ perceptions (27).

### 2.5 IPA procedure

Originally proposed by Martilla and James (33), IPA evaluates issues simultaneously across two dimensions: importance and performance. This method has been widely applied in various fields, including nursing education, healthcare quality assessment, and health education (34–36). In this study, IPA was used to identify management gaps among the risk factors perceived by shared nurses.

The importance axis (x-axis) was represented by the global weights derived from the CFPR results. The performance axis (y-axis) was represented by the mean scores of participants’ ratings regarding the current status of each risk factor in shared nursing practice, measured on a 10-point Likert scale. The four-quadrant framework is illustrated in Figure 1. The overall mean values of importance and performance served as the intersection point, dividing the matrix into four quadrants:

**Figure 1:**
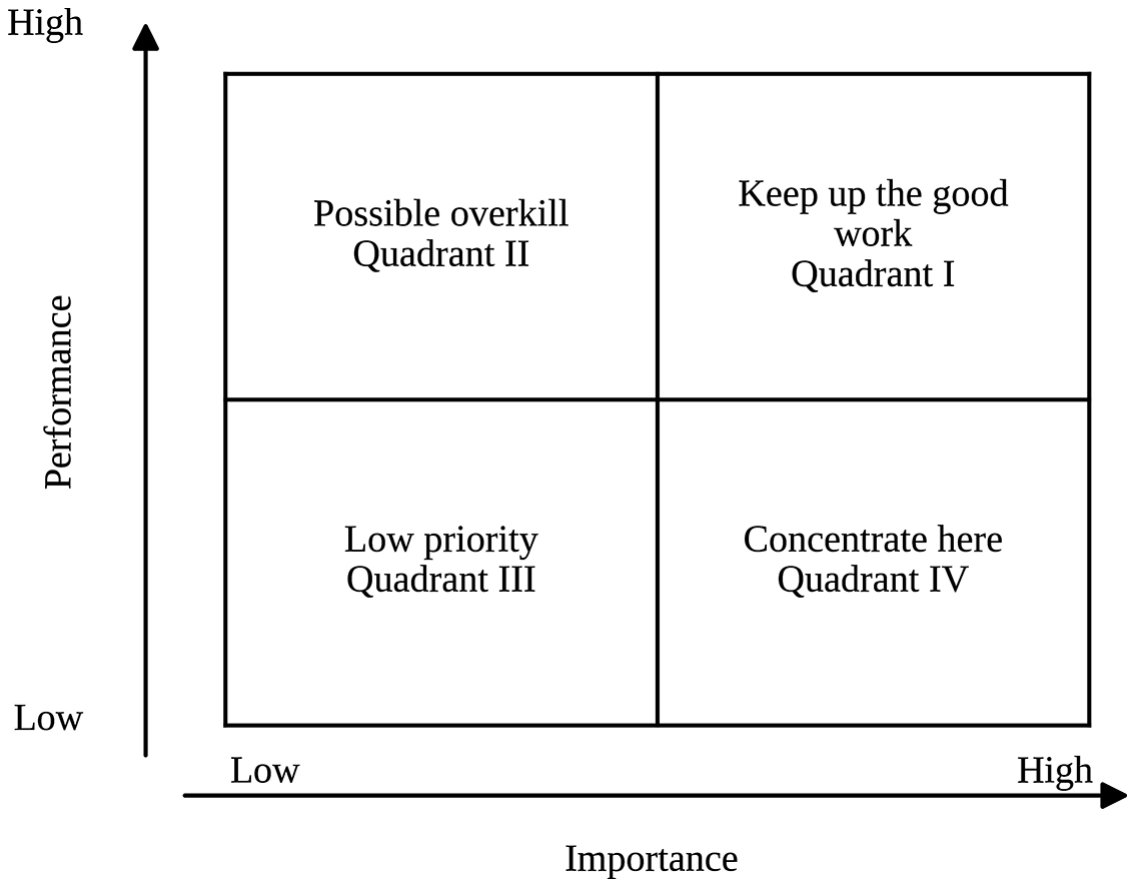
The four-quadrant diagram of importance–performance analysis. The x-axis represents importance and the y-axis represents performance. Quadrant I, high importance–high performance (keep up the good work); Quadrant II, low importance–high performance (possible overkill); Quadrant III, low importance–low performance (low priority); Quadrant IV, high importance–low performance (concentrate here).

Quadrant I: High importance–high performance (keep up the good work). This quadrant represents key risk factors that are highly important and are currently managed effectively. Existing strategies should be maintained and continuously strengthened to sustain performance.

Quadrant II: Low importance–high performance (possible overkill). This quadrant indicates potential resource over-allocation. It encompasses secondary factors that currently receive substantial investment. Resources allocated here should be redirected to higher-priority areas.

Quadrant III: Low importance–low performance (low priority). This quadrant comprises secondary factors of relatively low importance where performance remains poor. Given their limited strategic significance, interventions may be delayed while resources are directed toward more critical issues.

Quadrant IV: High importance–low performance (concentrate here). This quadrant contains factors that are highly important but currently managed poorly. Therefore, immediate interventions and improvements are required.

### 2.6 Data collection and survey procedure

The questionnaire survey was conducted in March 2026. Paper-based questionnaires were distributed face-to-face to eligible shared nurses by the research team at the study hospital. Before distribution, the investigators explained the purpose of the study and obtained verbal informed consent, as approved by the ethics committee. Participants completed the questionnaires independently and anonymously, and the documents were collected on site immediately after completion.

The questionnaire consisted of four sections. Section A presented the 27 risk items of the IBRF scale, grouped into five dimensions (C1–C5). In this section, participants rated the importance of each risk item using a 5-point Likert scale (1 = very unimportant, 5 = very important). This section served to familiarize participants with the risk-factor framework and to provide a preliminary importance ranking; it was not used to calculate the CFPR weights. Section B contained the CFPR pairwise-comparison task described above (see CFPR procedure). Section C assessed current management performance. Participants rated the current status of each of the 27 risk items using a 10-point Likert scale. The mean performance scores served as the performance axis in IPA, while the CFPR global weights served as the importance axis. Section D collected the participants’ sociodemographic and professional characteristics.

Upon collection, each questionnaire was checked for completeness; those with logically inconsistent answers or identical responses across all items were considered invalid and excluded from the analysis. A total of 33 questionnaires were distributed and returned. After excluding three invalid questionnaires, 30 valid responses were included, yielding a valid response rate of 90.91%.

### 2.7 Ethical considerations

This study was approved by the Medical Ethics Review Committee of Taizhou First People’s Hospital (approval number: 2025-KY102-01) and conducted in accordance with the Declaration of Helsinki. Before questionnaire distribution, the investigators explained the study purpose and procedures, and verbal informed consent was obtained from all participants, as approved by the ethics committee; participation was voluntary, and withdrawal was permitted at any time without consequence. Data were collected anonymously using paper-based questionnaires, with no personally identifiable information recorded. Completed questionnaires were stored in a locked cabinet accessible only to the research team, and the entered data were maintained on a password-protected computer; only aggregated results are reported.

## 3 Results

A total of 30 shared nurses who met the inclusion criteria were enrolled in this study, 90.00% (*n* = 27) of whom were women. The majority of participants were aged 31–40 years (66.67%, *n* = 20). Most participants held a bachelor’s degree (96.67%, *n* = 29). Regarding professional rank, 76.67% (*n* = 23) held an intermediate title, and 73.33% (*n* = 22) had 11–20 years of nursing experience. Participants were employed across various clinical departments, including internal medicine (30.00%, *n* = 9), surgery (23.33%, *n* = 7), emergency and intensive care (16.67%, *n* = 5), obstetrics and gynecology (10.00%, *n* = 3), pediatrics/neonatology (6.67%, *n* = 2), and others (13.33%, *n* = 4). The mean duration of experience providing home-based shared nursing services was 4.8 ± 1.94 years, with 40.00% (*n* = 12) of participants having cumulatively completed more than 30 service orders. Detailed participant characteristics are presented in Table 2.

**Table 2:** Background and characteristics (*n* = 30).

Characteristics	*n* (%)/Mean±SD
Gender	
Man	3 (10.00%)
Women	27 (90.00%)
Age	
31–40	20 (66.67%)
41–50	9 (30.00%)
≥ 51	1 (3.33%)
Education	
Junior college degree	1 (3.33%)
Bachelor’s degree	29 (96.67%)
Professional title	
Primary	3 (10.00%)
Intermediate	23 (76.67%)
Senior	4 (13.33%)
Years of nursing experience	
6–10	1 (3.33%)
11–20	22 (73.33%)
≥ 21	7 (23.33%)
Cumulative number of completed shared nursing service orders	
≤ 5	9 (30.00%)
6–15	7 (23.33%)
16–30	2 (6.67%)
≥ 31	12 (40.00%)
Department	
Surgery	7 (23.33%)
Internal Medicine	9 (30.00%)
Emergency and Intensive Care Unit	5 (16.67%)
Obstetrics and Gynecology	3 (10.00%)
Pediatrics/Neonatology	2 (6.67%)
Others	4 (13.33%)
Duration of shared nursing service experience (years)	4.8 ± 1.94

### 3.1 CFPR weight results

Risk factor weights were calculated using the CFPR method based on pairwise comparison judgments provided by the 30 shared nurses, who served as the expert panel. The detailed results are presented in Table 3. The consistency analysis revealed that the confidence level of the target layer was 99.22%, and the gap level was 0.78 (< 5%). The statistical confidence levels for each dimension ranged from 98.65 to 99.72% (all > 95%), indicating that the judgments of these 30 experts had good logical consistency and that the weight results were reliable.

**Table 3:** Weights of the risk factors derived from consistent fuzzy preference relations analysis.

Dimension	Local weight	Rank	Criteria	Local weight	Rank	Global weight	Rank
C1	0.225	2	C11	0.200	3	0.045	10
			C12	0.197	4	0.044	11
			C13	0.318	1	0.071	1
			C14	0.285	2	0.064	2
C2	0.147	5	C21	0.103	8	0.015	27
			C22	0.115	7	0.017	26
			C23	0.126	4	0.019	23
			C24	0.116	6	0.017	25
			C25	0.138	2	0.020	21
			C26	0.126	3	0.019	22
			C27	0.122	5	0.018	24
			C28	0.155	1	0.023	19
C3	0.190	4	C31	0.176	3	0.033	16
			C32	0.158	5	0.030	18
			C33	0.197	1	0.037	14
			C34	0.174	4	0.033	17
			C35	0.119	6	0.023	20
			C36	0.177	2	0.034	15
C4	0.198	3	C41	0.223	4	0.044	12
			C42	0.238	3	0.047	9
			C43	0.297	1	0.059	3
			C44	0.242	2	0.048	8
C5	0.240	1	C51	0.201	3	0.048	6
			C52	0.200	4	0.048	7
			C53	0.161	5	0.039	13
			C54	0.236	1	0.057	4
			C55	0.202	2	0.049	5

The statistical significance confidence levels (%) and average gap levels (%) were 99.22/0.78 for the goal layer, 99.03/0.97 for C1, 98.92/1.08 for C2, 99.40/0.60 for C3, 98.65/1.35 for C4, and 99.72/0.28 for C5. C1–C5 denote the five risk dimensions: C1, Patient/Family Risk Assessment; C2, External Environment Risk Assessment; C3, Platform/Hospital Supply Risk Assessment; C4, Nurses’ Service-related Experience; C5, Nurses’ Clinical Core Competence Assessment. C11–C55 denote the 27 risk items; full names are provided in Table 1.

At the dimensional level, the local weights ranked the five risk dimensions in the following order: Nurses’ Clinical Core Competence Assessment (C5), Patient/Family Risk Assessment (C1), Nurses’ Service-related Experience (C4), Platform/Hospital Supply Risk Assessment (C3), and External Environment Risk Assessment (C2).

At the item level, the five risk factors with the highest global weights were the establishment of a trust relationship with patients/family members (C13, 0.071), the consistency of service outcomes with the expectations of patients/family members (C14, 0.064), the adequacy of systematic and comprehensive home-care training (C43, 0.059), proficiency in accurately assessing clinical problems and performing emergency handling (C54, 0.057), and the ability to remain calm and orderly when dealing with difficult clinical problems (C55, 0.049).

### 3.2 IPA evaluation results

Based on the weights derived from the CFPR analysis, IPA was conducted to jointly evaluate the importance and performance of the risk factors. The IPA matrix was constructed using the mean importance value (w = 0.037) and the mean performance score (*p* = 7.612), resulting in the classification of the 27 risk factors into four quadrants. The results are summarized in Table 4, and a matrix quadrant diagram is presented in Figure 2. Classification was based on unrounded values: risk factors with importance or performance values greater than or equal to the corresponding mean were assigned to the high side. Notably, the unrounded global weight of C33 (0.0374) slightly exceeded the mean importance (0.0370), placing it in Quadrant I despite its rounded value (0.037) appearing equal to the mean in Table 4.

**Table 4:** Importance–performance analysis of the 27 risk factors.

Criteria	Importance	Performance	Quadrant
C11	0.045	6.367	IV
C12	0.044	6.033	IV
C13	0.071	7.433	IV
C14	0.064	7.900	I
C21	0.015	5.633	III
C22	0.017	6.067	III
C23	0.019	6.667	III
C24	0.017	6.200	III
C25	0.020	6.267	III
C26	0.019	6.333	III
C27	0.018	6.167	III
C28	0.023	7.033	III
C31	0.033	8.467	II
C32	0.030	8.100	II
C33	0.037	8.300	I
C34	0.033	8.367	II
C35	0.023	7.667	II
C36	0.034	8.467	II
C41	0.044	7.900	I
C42	0.047	8.100	I
C43	0.059	8.800	I
C44	0.048	8.433	I
C51	0.048	8.900	I
C52	0.048	9.033	I
C53	0.039	8.633	I
C54	0.057	9.167	I
C55	0.049	9.100	I
Center	0.037	7.612	

C11–C55 denote the 27 risk items; full names are provided in Table 1. Importance values are global weights derived from CFPR; performance values are mean scores on a 10-point Likert scale. Quadrants: I, high importance–high performance; II, low importance–high performance; III, low importance–low performance; IV, high importance–low performance.

**Figure 2:**
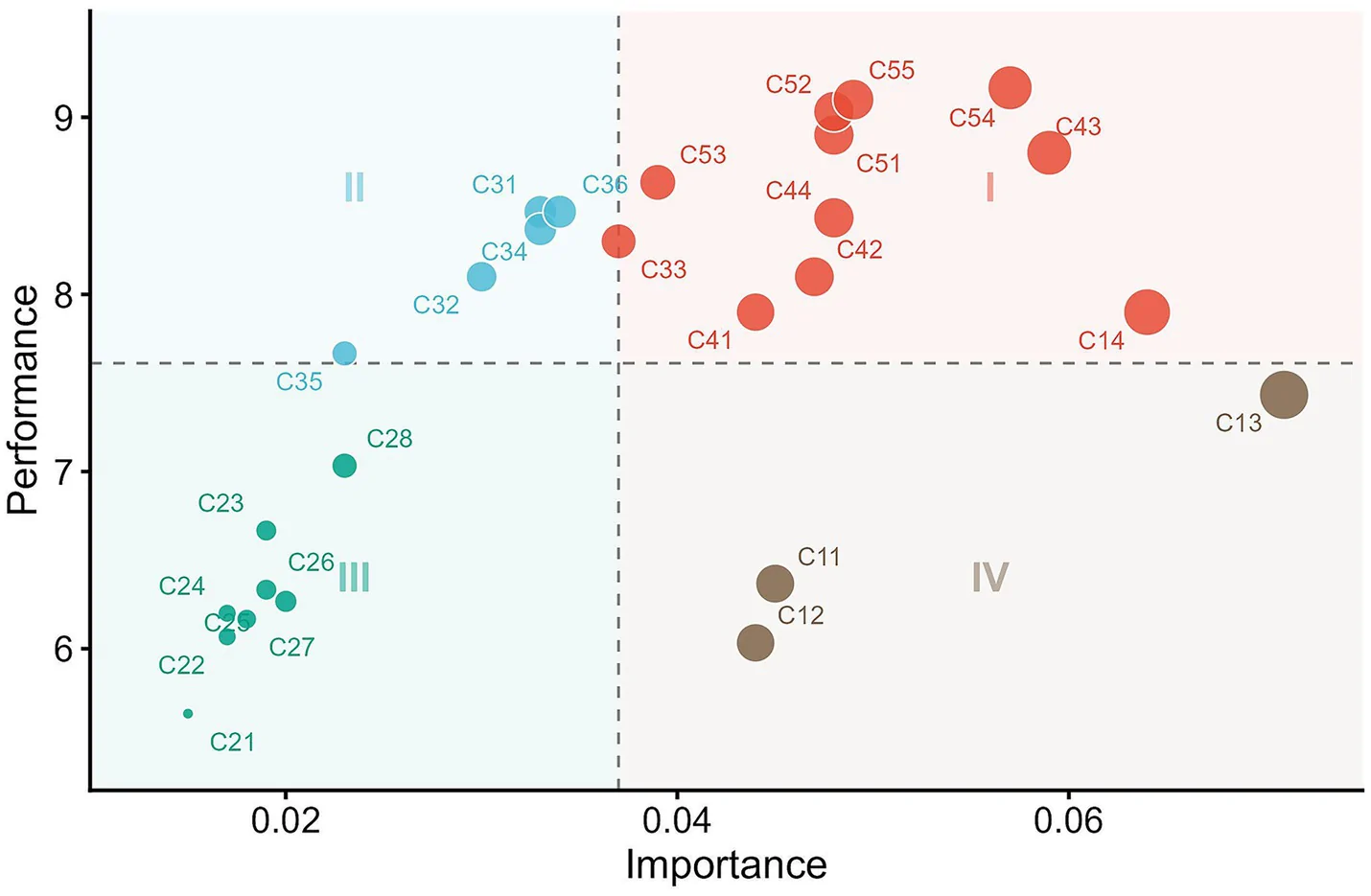
Importance–performance analysis matrix of the 27 risk factors in the study hospital. Each point represents one risk factor. The x-axis represents importance, operationalized as the global weight derived from the CFPR analysis, and the y-axis represents performance, operationalized as the mean score of the 10-point Likert ratings. The dashed lines indicate the overall means of importance (w = 0.037) and performance (*p* = 7.612), which divide the matrix into Quadrants I–IV. C11–C55 denote the risk factor items, whose full names are listed in Table 1.

Quadrant I contained 11 risk factors, namely C14, C33, C41–C44, and C51–C55 (full item names are listed in Table 1). These factors were considered highly important and were currently managed effectively, indicating that existing management strategies in these areas should be maintained and further strengthened.

Quadrant II included C31, C32, C34–C36. These factors demonstrated satisfactory performance despite their relatively lower importance, suggesting that resource allocation in these areas may exceed their strategic priority.

Quadrant III included C21–C28. Although performance levels for these factors were relatively low, their perceived importance was also limited, indicating that they may represent lower-priority areas for intervention under resource constraints.

Quadrant IV included three risk factors, all belonging to the Patient/Family Risk Assessment dimension: patient/family courtesy (C11), patient/family educational background (C12), and the establishment of trust with patients/family members (C13). These factors were identified as highly important but inadequately managed, and therefore, they represent priority targets for risk mitigation and management interventions.

## 4 Discussion

### 4.1 Key risk factors

At the dimension level, the three highest-ranked dimensions were Nurses’ Clinical Core Competence Assessment (C5), Patient/Family Risk Assessment (C1), and Nurses’ Service-related Experience (C4). These findings suggest that the key risk factors associated with shared nursing services extend beyond nurses’ professional competencies and service experience to include factors related to patient–family interactions.

After accepting service requests through the platform, shared nurses typically travel to patients’ homes independently or in pairs to provide nursing care. Operating away from the hospital’s professional environment, this model not only suffers from personnel constraints but also precludes timely team assistance during emergencies. Furthermore, it exposes nurses to diverse and unpredictable home environments (37). Experts considered professional nursing knowledge, clinical skills, independent decision-making capacity, and emergency response competence to be fundamental components of risk prevention. Accordingly, Nurses’ Clinical Core Competence Assessment (C5) received the highest weight. This finding is consistent with previous studies indicating that nurses’ core clinical competence not only influences service quality but also directly affects patient safety (38).

The high weighting assigned to Patient/Family Risk Assessment (C1) may be attributable to the unique characteristics of the shared nursing model. Unlike in hospital settings, where long-term therapeutic relationships are often established, shared nurses frequently provide care to unfamiliar patients in home environments (39). Although patients and their families voluntarily choose shared nursing services, cognitive biases or skepticism may exist regarding this relatively new service model. Previous studies have reported that some patients and family members perceive shared nursing as insufficiently standardized and potentially risky, resulting in lower levels of trust and less effective nurse–patient communication. Such challenges may adversely affect service quality and patient safety while simultaneously increasing occupational and personal safety risks for shared nurses (40).

Nurses’ Service-related Experience (C4) ranked third among the five dimensions, highlighting the importance of practical experiences in the risk management of shared nursing services. Shared nurses must adapt to diverse patient needs, lifestyles, and home environments, all of which vary substantially across service encounters. However, many nurses lack sufficient practical and training experience (41). Therefore, high-quality training and the accumulation of clinical experience are essential for enhancing nurses’ ability to identify potential risks, ensure patient safety, and respond effectively to unexpected situations encountered during service delivery.

Analysis of the global item weights showed that the top five highest-ranked risk factors were the establishment of a trust relationship with patients/family members (C13), the consistency of service outcomes with the expectations of patients/family members (C14), the adequacy of systematic and comprehensive home-care training (C43), proficiency in accurately assessing clinical problems and performing emergency handling (C54), and the ability to remain calm and orderly when dealing with difficult clinical problems (C55). These five risk factors not only correspond to the three highest-ranked dimensions but also illustrate the dynamic risk-management pathway throughout the delivery of shared nursing services. From the experts’ perspective, the adequacy of systematic training (C43) serves as a foundational component of service preparation and directly influences nurses’ clinical competence and performance during service delivery (C54 and C55). In turn, nurses’ clinical competence affects their ability to establish trust with patients and family members and to meet service expectations (C13 and C14). The quality of these interactions subsequently dictates the overall safety and outcomes of shared nursing services.

C43 ranked third in the global weights, reflecting the experts’ emphasis on preparatory work for shared nursing services. Although shared nurses undergo rigorous qualification assessments before providing services, the dynamic nature of home-care environments and the unique characteristics of shared nursing practice may create competency requirements that extend beyond conventional clinical training. Consequently, targeted and practice-oriented training programs are essential to ensure service quality and safety. Such programs could combine simulated home-visit scenarios, emergency handling drills, and communication skills training delivered in blended online–offline formats, with a competency assessment required before nurses undertake independent home-based assignments, followed by periodic recertification. Guo et al. (23) suggested that educational activities, including knowledge-based lectures and training programs related to shared nursing services, can enhance nurses’ risk awareness and improve service quality.

While training represents an important upstream component of risk management, its value ultimately depends on nurses’ ability to translate knowledge into effective clinical practice. This interpretation is supported by the high weights assigned to C54 and C55. These two indicators further characterize the multidimensional nature of nurses’ core clinical competence, encompassing knowledge, skills, attitudes, behaviors, and psychological qualities. Specifically, they reflect nurses’ ability to identify potential risks to both patients and themselves, promptly observe and analyze changes in patients’ conditions, maintain a calm and decisive mindset, and skillfully perform emergency interventions. The present findings underscore the importance of nurses’ competence in maintaining patient safety during home-based service delivery. Previous studies have similarly reported that the competence of shared nurses is critical to service quality (42) and constitutes a key component of risk management in home-care settings (43).

C13 and C14, which ranked first and second in the global weights, respectively, highlight the central role of nurse–patient interactions in shared nursing services. Demand for home-based nursing care is often driven by conditions such as mobility limitations and chronic illnesses, leading patients and family members to place high expectations on service convenience, accessibility, and effectiveness. However, constraints in staffing and healthcare resources may limit the extent to which these expectations can be met. Bu et al. noted that patients and their families are concerned about the safety of home care services (44) and that the establishment of trust between nurses and patients can be challenging. The degree of trust placed in nurses directly influences their psychological well-being and the efficiency of service delivery (45). Therefore, nurse–patient communication and trust-building should be regarded as fundamental components of risk management in shared nursing services. Concretely, healthcare institutions and platform operators could develop targeted communication training modules for shared nurses, incorporating scenario-based simulations of initial home visits, structured protocols for aligning service expectations with patients and families through the platform’s messaging system before service delivery, and guided rehearsals of responses to common difficult interactions. Complementing these nurse-side measures, hospitals could implement a structured onboarding session for new patients and their families before the initial home visit—introducing the assigned nurse, explaining the service model and procedures, and setting mutual expectations—supplemented by informational brochures and short educational videos accessible through the platform.

### 4.2 Key improvement gaps

According to the IPA results, factors located in Quadrant IV are considered highly important but insufficiently addressed in current practice. These factors represent priority areas requiring immediate managerial attention and targeted interventions. In the present study, Quadrant IV contained three risk factors, all of which belonged to the Patient/Family Risk Assessment (C1) dimension: patient/family courtesy (C11), patient/family educational background (C12), and the establishment of trust with patients/family members (C13). Ranked by global weight, C13 was the most important factor, followed by C11 and C12. Notably, factors such as patient and family courtesy, educational background, and personal conduct are largely beyond the direct control of healthcare organizations in the short term. Similarly, the establishment of a trusting nurse–patient relationship depends on the active participation of both nurses and service recipients. Therefore, the inability to directly control patient and family behaviors contributes to a lack of established management practices in this area, making these risk factors critical targets for managerial intervention.

Although shared nursing services have been implemented in China for several years, public awareness of this emerging medical care model is lower than expected (46), and the acceptance rate remains low (39). To address this gap, healthcare institutions and platform operators should strengthen public education and promotion efforts through diversified communication channels. For younger populations, Internet-based media represents an effective means of disseminating information about shared nursing services (44). Given the information-saturated online environment, promotional materials should be designed in a clear, accessible, and engaging manner. For example, service information could be presented through visual formats such as animated demonstrations, infographics, and short educational videos to improve understanding and enhance trust in the service model. In contrast, older adults tend to use online platforms less frequently and may exhibit greater skepticism toward digital health services (47). Therefore, healthcare institutions should complement online promotion with face-to-face activities, such as community lectures and free medical consultations. Furthermore, given the localized nature of shared healthcare services, institutions may consider introducing shared nurses to the community during promotional activities by detailing their professional qualifications, clinical experience, and service scope. Such measures may facilitate the early development of familiarity and trust before service delivery, thereby reducing uncertainty during initial encounters. Additionally, managers should improve industry standards for shared healthcare services, clarify service provision procedures, enhance nurses’ communication skills, and cultivate their ability to actively build trusting relationships.

C11 and C12 primarily reflect the characteristics and behavioral attributes of patients and their family members. Workplace violence is a well-documented occupational hazard in home-based nursing. In a survey of home visiting nurses at public health centers in Korea, 70.6% reported having experienced workplace violence (48); in China, a cross-sectional study of 2,769 hospital nurses found that 49.12% had experienced at least one type of workplace violence within the past 6 months (49). Although comparable data specific to Chinese shared nurses are not yet available, their practice model—independent home visits in non-institutional settings—may further amplify interpersonal safety risks. Therefore, risk management efforts should focus not solely on changing patient or family behaviors but also on strengthening protective measures for nurses. Healthcare institutions should establish comprehensive safety support mechanisms, create psychological care groups, and provide targeted mental health support to nurses who encounter interpersonal conflicts. In addition, for situations involving difficult communication or challenging patient and family attitudes, managers may organize regular case review meetings and encourage experienced nurses to share effective coping strategies and communication techniques. Furthermore, platform operators should consider implementing a risk warning system. For example, users with documented inappropriate or abusive behaviors could be flagged within the platform, generating risk alerts for nurses before they accept service assignments. Such measures may assist nurses in conducting risk assessments and making informed decisions regarding service provision.

### 4.3 Limitations

This study has several limitations. First, the study relied on a small, single-center expert panel of 30 shared nurses from one comprehensive tertiary hospital in Zhejiang Province. Although a panel of this size is consistent with common practice in MCDM- and CFPR-based studies, the modest sample size may limit the precision and stability of the derived weights. Furthermore, the single-center design may limit the generalizability of the findings to other regions and institutional settings, even though Zhejiang is a pilot region for shared nursing services and the experts possessed substantial frontline experience. Therefore, the findings should be interpreted as exploratory. Because nurses from community health centers are increasingly participating in shared nursing services, future multicenter studies with larger and more diverse expert panels are needed to validate and refine the identified weights and improvement priorities. Second, the risk factor weights and the IPA were derived from the subjective judgments of an expert panel, which may introduce perception or self-report bias. To validate and refine the present findings, future studies should triangulate these expert-based priorities with objective outcome data—such as adverse event reports, complaint records, and platform service logs—as well as with the perspectives of patients and their families, who may perceive and prioritize risks differently. Third, data were collected at a single time point and therefore reflect only the contemporaneous perceptions of participating experts. Given the rapid development of shared nursing services and the evolving healthcare environment, risk factor perceptions may change over time. Future research could employ longitudinal designs or time-series analyses to examine the dynamic evolution of risk factors and to validate the stability of the identified priorities.

## 5 Conclusion

Based on the IBRF scale, this study adopted a hybrid MCDM model integrating CFPR and IPA to systematically identify the key risk factors and their improvement priorities for shared nursing services in the study hospital. Nurses’ clinical core competence (including problem-solving, emergency handling, psychological resilience, and reflective ability), patient/family risk assessment (including trust relationships and expectation levels), and nurses’ service-related experience (primarily training received) constituted the most important risk dimensions from an expert perspective. The IPA results further revealed that patient/family courtesy, educational background, and the establishment of trust represent high-priority areas with relatively inadequate management performance. These findings suggest that risk management in similar settings may be advanced through concrete strategies, including developing targeted communication training modules for shared nurses, implementing a platform-based risk alert system that flags users with documented inappropriate behavior before service assignment, launching public awareness campaigns through both digital and community-based channels tailored to different age groups, and establishing standardized service protocols alongside safety support mechanisms for nurses. Given the single-center design and the modest sample of 30 experts, these findings should be considered preliminary and validated in future multicenter studies with larger, more diverse samples before being generalized to shared nursing services across China.

## Data Availability

The original contributions presented in the study are included in the article/Supplementary material, further inquiries can be directed to the corresponding author.
