# Supplementary material for: Identifying and prioritizing key risk factors in internet-based shared nursing services from the nurses’ perspective: a hybrid multi-criteria decision-making approach
Source: Front Public Health. 2026 Oct 19;14:1956352. doi: 10.3389/fpubh.2026.1956352 (PMC13724534; doi:10.3389/fpubh.2026.1956352)
Supplement: Supplementary file 1 [file Data_Sheet_1.docx]

Supplementary Material

# 1 Supplementary Tables S1: Questionnaire

**Questionnaire for the Study on Risk Factor Analysis of "Internet+ Nursing Services"**

**Questionnaire No.:** ________________

Dear nursing colleague,

Thank you very much for taking the time to participate in our survey. We sincerely invite you to complete this questionnaire for the study on risk factor analysis of "Internet+ Nursing Services." This study aims to collect relevant information, provide targeted risk countermeasures for nurses participating in "Internet+ Nursing Services," and strengthen risk management.

# [Informed Consent — Confirmation of Oral Informed Consent]

Before completing the questionnaire, please read the following information carefully and confirm whether you understand the purpose and content of this study and orally agree to participate:

1. Purpose of the study: This study aims to comprehensively identify the key risk factors of "Internet+ Nursing Services," provide targeted risk countermeasures for participating nurses, and strengthen risk management.

2. Study procedure: You will be asked to complete a questionnaire containing multiple questions. The estimated completion time is 10–30 minutes.

3. Confidentiality: Your answers will be kept confidential. All data will be processed anonymously and your personal information will not be disclosed.

4. Voluntariness: Participation in this questionnaire survey is entirely voluntary. You have the right to discontinue participation at any time without giving a reason.

5. Risks and inconvenience: There are no known risks from participating in this study, but it may take approximately 10–30 minutes of your time.

6. Contact information: If you have any questions or difficulties during the study, please contact us directly. Contact person: ____________ Telephone: ____________

If you have read and understood the above information and agree to participate in this study, please tick the box below as proof of oral consent, and then continue completing the questionnaire.

☐ I have read and understood the above information, and I also understand that I may withdraw from this study at any time while completing it. I agree to participate in this study and complete the following questionnaire; ticking this option serves as confirmation of oral informed consent.

If you decide not to participate, we respect your decision and no inconvenience will result. Thank you again for considering participation in our questionnaire survey.

# Risk Factor Framework for "Internet+ Nursing Services"

| Dimension | Criterion / Element |
| --- | --- |
| C1: Patient/Family Risk Assessment | C11: Patient/family courtesy |
|  | C12: Patient/family educational background |
|  | C13: Establishment of a trust relationship with patients/family members |
|  | C14: Consistency of service outcomes with the expectations of patients/family members |
| C2: External Environment Risk Assessment | C21: Favorability of weather on the service day |
|  | C22: Appropriateness of the distance between the service location and the departure point |
|  | C23: Safety of transportation on the service day |
|  | C24: Condition of roads and convenience of transportation on the service day |
|  | C25: Accessibility of the residential community and convenience of surrounding transportation |
|  | C26: Security and safety of the residential community environment |
|  | C27: Safety of the client's home environment (e.g., unobstructed passages, non-slip floors) |
|  | C28: Adequacy of lighting in the client’s home and nursing operation environment |
| C3: Platform/Hospital Supply Risk Assessment | C31: Provision of effective supplies within their shelf life by the platform/hospital |
|  | C32: Provision of work recorders by the platform/hospital to ensure service transparency |
|  | C33: Provision of app-based location tracking and one-click alarm devices by the platform/hospital |
|  | C34: Information and data security of the platform/hospital |
|  | C35: Reasonableness of service pricing by the platform/hospital |
|  | C36: Provision of medical liability and personal accident insurance for nurses by the platform/hospital |
| C4: Nurses' Service-related Experience | C41: Institutional encouragement of Internet Plus Nursing Services home visits |
|  | C42: Understanding of qualification requirements and relevant laws and regulations |
|  | C43: Adequacy of systematic and comprehensive home-care training |
|  | C44: Level of experience in Internet Plus Nursing Services (≥3 participations) |
| C5: Nurses' Clinical Core Competence Assessment | C51: Alignment of professional knowledge with service requirements |
|  | C52: Proficiency in nursing operation skills |
|  | C53: Effectiveness of communication with patients and their families |
|  | C54: Proficiency in accurately assessing clinical problems and performing emergency handling |
|  | C55: Ability to remain calm and orderly when dealing with difficult clinical problems |

# Section A: Importance Assessment Table

*Importance score: 1 = very unimportant; 5 = very important.*

| Dimension | Criterion / Element | 1 | 2 | 3 | 4 | 5 |
| --- | --- | --- | --- | --- | --- | --- |
| C1. Patient/Family-Related Risk Assessment | C11. The patient/family are polite and courteous to me. |  |  |  |  |  |
|  | C12. The patient/family have a relatively high educational level, good quality, and good self-cultivation. |  |  |  |  |  |
|  | C13. The patient/family have established a trusting relationship with me. |  |  |  |  |  |
|  | C14. The service effect is basically consistent with the expectations of the family/patient. |  |  |  |  |  |
| C2. External Environment Risk Assessment | C21. The weather on the service day is clear and suitable for travel, with no severe weather such as snow, fog, or strong wind. |  |  |  |  |  |
|  | C22. The distance between the service location and the departure point is appropriate. |  |  |  |  |  |
|  | C23. The transportation taken on the service day is safe. |  |  |  |  |  |
|  | C24. Road conditions are smooth on the service day and transportation is convenient. |  |  |  |  |  |
|  | C25. The community where the service location is situated is easy to find, and nearby transportation is convenient. |  |  |  |  |  |
|  | C26. The service community has sound security and a safe environment. |  |  |  |  |  |
|  | C27. The service recipient's home environment is safe, with no clutter blocking passage, overly slippery floors, or similar hazards. |  |  |  |  |  |
|  | C28. The service recipient's home and the nursing-operation environment have sufficient lighting. |  |  |  |  |  |
| C3. Platform/Hospital Supply Risk Assessment | C31. The Internet platform/hospital can provide effective supplies within their shelf life. |  |  |  |  |  |
|  | C32. The Internet platform/hospital can provide work-recording devices, such as video or photographic equipment, to keep the whole service process transparent. |  |  |  |  |  |
|  | C33. The Internet platform/hospital can provide a mobile-app location-tracking system and devices such as one-click alarms and delayed-warning devices to protect my personal safety. |  |  |  |  |  |
|  | C34. The Internet platform/hospital keeps information and data secure, and personal information is not easily disclosed. |  |  |  |  |  |
|  | C35. The Internet platform/hospital prices services reasonably. |  |  |  |  |  |
|  | C36. The Internet platform/hospital purchases medical liability insurance and personal accident insurance for nurses. |  |  |  |  |  |
| C4. Nurse Service-Related Experience | C41. My medical institution encourages nurses to provide home-based "Internet+ Nursing Services." |  |  |  |  |  |
|  | C42. I understand the entry qualifications for "Internet+ Nursing Services" nurses and the relevant laws and regulations. |  |  |  |  |  |
|  | C43. I have received systematic and comprehensive home-care training, including relevant "Internet+ Nursing Services" policies and regulations, management of difficult nursing problems, emergency nursing response, interpersonal communication, and professional competence. |  |  |  |  |  |
|  | C44. I have rich experience in "Internet+ Nursing Services" (participation in "Internet+ Nursing Services" ≥ 3 times). |  |  |  |  |  |
| C5. Assessment of Nurses' Clinical Core Competence | C51. My professional knowledge reserve closely matches the knowledge required for this service item. |  |  |  |  |  |
|  | C52. My nursing operation skills are highly proficient. |  |  |  |  |  |
|  | C53. I communicate well with patients and their families. |  |  |  |  |  |
|  | C54. I can correctly assess clinical problems, solve practical nursing problems, provide health education, and respond to emergencies. |  |  |  |  |  |
|  | C55. When dealing with difficult clinical problems, I can remain calm and methodical, engage quickly, and summarize experience effectively. |  |  |  |  |  |

# Section B: Relative Preference Among Risk Factors for "Internet+ Nursing Services"

## Instructions and Example

[Part 1] Importance ranking: For "risk factors of Internet+ Nursing Services," how would you rank the importance of the following indicators with respect to nurses' sensitivity in nursing? Example: I think the importance ranking of the evaluation indicators for "risk factors of Internet+ Nursing Services" is C1 > C2 > C3 > C4 > C5.

| Goal | Indicator | Importance rank |
| --- | --- | --- |
| Risk factor assessment for "Internet+ Nursing Services" | C1 Patient/Family-Related Risk Assessment | ________________ |
| Risk factor assessment for "Internet+ Nursing Services" | C2 External Environment Risk Assessment | ________________ |
| Risk factor assessment for "Internet+ Nursing Services" | C3 Platform/Hospital Supply Risk Assessment | ________________ |
| Risk factor assessment for "Internet+ Nursing Services" | C4 Nurse Service-Related Experience | ________________ |
| Risk factor assessment for "Internet+ Nursing Services" | C5 Assessment of Nurses' Clinical Core Competence | ________________ |

*Note: If indicators are considered equally important, the same rank may be assigned. This is only for preliminary reflection on differences in indicator importance and is not mandatory.*

[Part 2] Importance quantification: Based on the importance ranking in Part 1, please compare the following indicators pairwise and mark the degree of agreement with "V."

## Meaning of the Evaluation Scale

| Scale | Definition | Explanation |
| --- | --- | --- |
| 1 | Equal importance | The two indicators are equally important. |
| 3 | Weak importance | There is slight reason to support one factor as slightly more important. |
| 5 | Essential importance | Good reasons and logical judgment show that one factor is essentially more important. |
| 7 | Very strong importance | Evidence shows that one factor is very strongly more important. |
| 9 | Absolute importance | Strong evidence affirms that one factor is absolutely more important. |
| 2, 4, 6, 8 | Intermediate values between adjacent judgments | Use when a judgment falls between two adjacent scale values. |

Simple understanding: compare the indicator on the left with the indicator on the right. The closer the mark is to the left, the more important the left indicator is than the right indicator; the closer the mark is to the right, the more important the right indicator is than the left indicator.

**=============== Begin Completion (please complete the highlighted areas) ===============**

## 1. For "risk factors of Internet+ Nursing Services," how should the following indicators be ranked? Start from 1; equal ranks may be used when importance is the same.

| Goal | Indicator | Importance rank |
| --- | --- | --- |
| Risk factor assessment for "Internet+ Nursing Services" | C1 Patient/Family-Related Risk Assessment | ________________ |
|  | C2 External Environment Risk Assessment | ________________ |
|  | C3 Platform/Hospital Supply Risk Assessment | ________________ |
|  | C4 Nurse Service-Related Experience | ________________ |
|  | C5 Assessment of Nurses' Clinical Core Competence | ________________ |

Based on the ranking above, compare the importance of the following indicators pairwise. Mark with "V."

| Indicator | Relative importance ratio | | | | | | | | | | | | | | | | | Indicator |
| --- | --- | --- | --- | --- | --- | --- | --- | --- | --- | --- | --- | --- | --- | --- | --- | --- | --- | --- |
|  | 9:1 | 8:1 | 7:1 | 6:1 | 5:1 | 4:1 | 3:1 | 2:1 | 1:1 | 1:2 | 1:3 | 1:4 | 1:5 | 1:6 | 1:7 | 1:8 | 1:9 |  |
| C1 Patient/Family-Related Risk Assessment |  |  |  |  |  |  |  |  |  |  |  |  |  |  |  |  |  | C2 External Environment Risk Assessment |
| C2 External Environment Risk Assessment |  |  |  |  |  |  |  |  |  |  |  |  |  |  |  |  |  | C3 Platform/Hospital Supply Risk Assessment |
| C3 Platform/Hospital Supply Risk Assessment |  |  |  |  |  |  |  |  |  |  |  |  |  |  |  |  |  | C4 Nurse Service-Related Experience |
| C4 Nurse Service-Related Experience |  |  |  |  |  |  |  |  |  |  |  |  |  |  |  |  |  | C5 Assessment of Nurses' Clinical Core Competence |

## 2. For C1 Patient/Family-Related Risk Assessment, how should the following indicators be ranked? Start from 1; equal ranks may be used when importance is the same.

| Goal | Indicator | Importance rank |
| --- | --- | --- |
| C1 Patient/Family-Related Risk Assessment | C11 The patient/family are polite and courteous to me. | ________________ |
|  | C12 The patient/family have a relatively high educational level, good quality, and good self-cultivation. | ________________ |
|  | C13 The patient/family have established a trusting relationship with me. | ________________ |
|  | C14 The service effect is basically consistent with the expectations of the family/patient. | ________________ |

Based on the ranking above, compare the importance of the following indicators pairwise. Mark with "V."

| Indicator | Relative importance ratio | | | | | | | | | | | | | | | | | Indicator |
| --- | --- | --- | --- | --- | --- | --- | --- | --- | --- | --- | --- | --- | --- | --- | --- | --- | --- | --- |
|  | 9:1 | 8:1 | 7:1 | 6:1 | 5:1 | 4:1 | 3:1 | 2:1 | 1:1 | 1:2 | 1:3 | 1:4 | 1:5 | 1:6 | 1:7 | 1:8 | 1:9 |  |
| C11 The patient/family are polite and courteous to me. |  |  |  |  |  |  |  |  |  |  |  |  |  |  |  |  |  | C12 The patient/family have a relatively high educational level, good quality, and good self-cultivation. |
| C12 The patient/family have a relatively high educational level, good quality, and good self-cultivation. |  |  |  |  |  |  |  |  |  |  |  |  |  |  |  |  |  | C13 The patient/family have established a trusting relationship with me. |
| C13 The patient/family have established a trusting relationship with me. |  |  |  |  |  |  |  |  |  |  |  |  |  |  |  |  |  | C14 The service effect is basically consistent with the expectations of the family/patient. |

## 3. For C2 External Environment Risk Assessment, how should the following indicators be ranked? Start from 1; equal ranks may be used when importance is the same.

| Goal | Indicator | Importance rank |
| --- | --- | --- |
| C2 External Environment Risk Assessment | C21 The weather on the service day is clear and suitable for travel, with no severe weather such as snow, fog, or strong wind. | ________________ |
|  | C22 The distance between the service location and the departure point is appropriate. | ________________ |
|  | C23 The transportation taken on the service day is safe. | ________________ |
|  | C24 Road conditions are smooth on the service day and transportation is convenient. | ________________ |
|  | C25 The community where the service location is situated is easy to find, and nearby transportation is convenient. | ________________ |
|  | C26 The service community has sound security and a safe environment. | ________________ |
|  | C27 The service recipient's home environment is safe, with no clutter blocking passage, overly slippery floors, or similar hazards. | ________________ |
|  | C28 The service recipient's home and the nursing-operation environment have sufficient lighting. | ________________ |

Based on the ranking above, compare the importance of the following indicators pairwise. Mark with "V."

| Indicator | Relative importance ratio | | | | | | | | | | | | | | | | | Indicator |
| --- | --- | --- | --- | --- | --- | --- | --- | --- | --- | --- | --- | --- | --- | --- | --- | --- | --- | --- |
|  | 9:1 | 8:1 | 7:1 | 6:1 | 5:1 | 4:1 | 3:1 | 2:1 | 1:1 | 1:2 | 1:3 | 1:4 | 1:5 | 1:6 | 1:7 | 1:8 | 1:9 |  |
| C21 The weather on the service day is clear and suitable for travel, with no severe weather such as snow, fog, or strong wind. |  |  |  |  |  |  |  |  |  |  |  |  |  |  |  |  |  | C22 The distance between the service location and the departure point is appropriate. |
| C22 The distance between the service location and the departure point is appropriate. |  |  |  |  |  |  |  |  |  |  |  |  |  |  |  |  |  | C23 The transportation taken on the service day is safe. |
| C23 The transportation taken on the service day is safe. |  |  |  |  |  |  |  |  |  |  |  |  |  |  |  |  |  | C24 Road conditions are smooth on the service day and transportation is convenient. |
| C24 Road conditions are smooth on the service day and transportation is convenient. |  |  |  |  |  |  |  |  |  |  |  |  |  |  |  |  |  | C25 The community where the service location is situated is easy to find, and nearby transportation is convenient. |
| C25 The community where the service location is situated is easy to find, and nearby transportation is convenient. |  |  |  |  |  |  |  |  |  |  |  |  |  |  |  |  |  | C26 The service community has sound security and a safe environment. |
| C26 The service community has sound security and a safe environment. |  |  |  |  |  |  |  |  |  |  |  |  |  |  |  |  |  | C27 The service recipient's home environment is safe, with no clutter blocking passage, overly slippery floors, or similar hazards. |
| C27 The service recipient's home environment is safe, with no clutter blocking passage, overly slippery floors, or similar hazards. |  |  |  |  |  |  |  |  |  |  |  |  |  |  |  |  |  | C28 The service recipient's home and the nursing-operation environment have sufficient lighting. |

## 4. For C3 Platform/Hospital Supply Risk Assessment, how should the following indicators be ranked? Start from 1; equal ranks may be used when importance is the same.

| Goal | Indicator | Importance rank |
| --- | --- | --- |
| C3 Platform/Hospital Supply Risk Assessment | C31 The Internet platform/hospital can provide effective supplies within their shelf life. | ________________ |
|  | C32 The Internet platform/hospital can provide work-recording devices, such as video or photographic equipment, to keep the whole service process transparent. | ________________ |
|  | C33 The Internet platform/hospital can provide a mobile-app location-tracking system and devices such as one-click alarms and delayed-warning devices to protect my personal safety. | ________________ |
|  | C34 The Internet platform/hospital keeps information and data secure, and personal information is not easily disclosed. | ________________ |
|  | C35 The Internet platform/hospital prices services reasonably. | ________________ |
|  | C36 The Internet platform/hospital purchases medical liability insurance and personal accident insurance for nurses. | ________________ |

Based on the ranking above, compare the importance of the following indicators pairwise. Mark with "V."

| Indicator | Relative importance ratio | | | | | | | | | | | | | | | | | Indicator |
| --- | --- | --- | --- | --- | --- | --- | --- | --- | --- | --- | --- | --- | --- | --- | --- | --- | --- | --- |
|  | 9:1 | 8:1 | 7:1 | 6:1 | 5:1 | 4:1 | 3:1 | 2:1 | 1:1 | 1:2 | 1:3 | 1:4 | 1:5 | 1:6 | 1:7 | 1:8 | 1:9 |  |
| C31 The Internet platform/hospital can provide effective supplies within their shelf life. |  |  |  |  |  |  |  |  |  |  |  |  |  |  |  |  |  | C32 The Internet platform/hospital can provide work-recording devices, such as video or photographic equipment, to keep the whole service process transparent. |
| C32 The Internet platform/hospital can provide work-recording devices, such as video or photographic equipment, to keep the whole service process transparent. |  |  |  |  |  |  |  |  |  |  |  |  |  |  |  |  |  | C33 The Internet platform/hospital can provide a mobile-app location-tracking system and devices such as one-click alarms and delayed-warning devices to protect my personal safety. |
| C33 The Internet platform/hospital can provide a mobile-app location-tracking system and devices such as one-click alarms and delayed-warning devices to protect my personal safety. |  |  |  |  |  |  |  |  |  |  |  |  |  |  |  |  |  | C34 The Internet platform/hospital keeps information and data secure, and personal information is not easily disclosed. |
| C34 The Internet platform/hospital keeps information and data secure, and personal information is not easily disclosed. |  |  |  |  |  |  |  |  |  |  |  |  |  |  |  |  |  | C35 The Internet platform/hospital prices services reasonably. |
| C35 The Internet platform/hospital prices services reasonably. |  |  |  |  |  |  |  |  |  |  |  |  |  |  |  |  |  | C36 The Internet platform/hospital purchases medical liability insurance and personal accident insurance for nurses. |

## 5. For C4 Nurse Service-Related Experience, how should the following indicators be ranked? Start from 1; equal ranks may be used when importance is the same.

| Goal | Indicator | Importance rank |
| --- | --- | --- |
| C4 Nurse Service-Related Experience | C41 My medical institution encourages nurses to provide home-based "Internet+ Nursing Services." | ________________ |
|  | C42 I understand the entry qualifications for "Internet+ Nursing Services" nurses and the relevant laws and regulations. | ________________ |
|  | C43 I have received systematic and comprehensive home-care training, including relevant "Internet+ Nursing Services" policies and regulations, management of difficult nursing problems, emergency nursing response, interpersonal communication, and professional competence. | ________________ |
|  | C44 I have rich experience in "Internet+ Nursing Services" (participation in "Internet+ Nursing Services" ≥ 3 times). | ________________ |

Based on the ranking above, compare the importance of the following indicators pairwise. Mark with "V."

| Indicator | Relative importance ratio | | | | | | | | | | | | | | | | | Indicator |
| --- | --- | --- | --- | --- | --- | --- | --- | --- | --- | --- | --- | --- | --- | --- | --- | --- | --- | --- |
|  | 9:1 | 8:1 | 7:1 | 6:1 | 5:1 | 4:1 | 3:1 | 2:1 | 1:1 | 1:2 | 1:3 | 1:4 | 1:5 | 1:6 | 1:7 | 1:8 | 1:9 |  |
| C41 My medical institution encourages nurses to provide home-based "Internet+ Nursing Services." |  |  |  |  |  |  |  |  |  |  |  |  |  |  |  |  |  | C42 I understand the entry qualifications for "Internet+ Nursing Services" nurses and the relevant laws and regulations. |
| C42 I understand the entry qualifications for "Internet+ Nursing Services" nurses and the relevant laws and regulations. |  |  |  |  |  |  |  |  |  |  |  |  |  |  |  |  |  | C43 I have received systematic and comprehensive home-care training, including relevant "Internet+ Nursing Services" policies and regulations, management of difficult nursing problems, emergency nursing response, interpersonal communication, and professional competence. |
| C43 I have received systematic and comprehensive home-care training, including relevant "Internet+ Nursing Services" policies and regulations, management of difficult nursing problems, emergency nursing response, interpersonal communication, and professional competence. |  |  |  |  |  |  |  |  |  |  |  |  |  |  |  |  |  | C44 I have rich experience in "Internet+ Nursing Services" (participation in "Internet+ Nursing Services" ≥ 3 times). |

## 6. For C5 Assessment of Nurses' Clinical Core Competence, how should the following indicators be ranked? Start from 1; equal ranks may be used when importance is the same.

| Goal | Indicator | Importance rank |
| --- | --- | --- |
| C5 Assessment of Nurses' Clinical Core Competence | C51 My professional knowledge reserve closely matches the knowledge required for this service item. | ________________ |
|  | C52 My nursing operation skills are highly proficient. | ________________ |
|  | C53 I communicate well with patients and their families. | ________________ |
|  | C54 I can correctly assess clinical problems, solve practical nursing problems, provide health education, and respond to emergencies. | ________________ |
|  | C55 When dealing with difficult clinical problems, I can remain calm and methodical, engage quickly, and summarize experience effectively. | ________________ |

Based on the ranking above, compare the importance of the following indicators pairwise. Mark with "V."

| Indicator | Relative importance ratio | | | | | | | | | | | | | | | | | Indicator |
| --- | --- | --- | --- | --- | --- | --- | --- | --- | --- | --- | --- | --- | --- | --- | --- | --- | --- | --- |
|  | 9:1 | 8:1 | 7:1 | 6:1 | 5:1 | 4:1 | 3:1 | 2:1 | 1:1 | 1:2 | 1:3 | 1:4 | 1:5 | 1:6 | 1:7 | 1:8 | 1:9 |  |
| C51 My professional knowledge reserve closely matches the knowledge required for this service item. |  |  |  |  |  |  |  |  |  |  |  |  |  |  |  |  |  | C52 My nursing operation skills are highly proficient. |
| C52 My nursing operation skills are highly proficient. |  |  |  |  |  |  |  |  |  |  |  |  |  |  |  |  |  | C53 I communicate well with patients and their families. |
| C53 I communicate well with patients and their families. |  |  |  |  |  |  |  |  |  |  |  |  |  |  |  |  |  | C54 I can correctly assess clinical problems, solve practical nursing problems, provide health education, and respond to emergencies. |
| C54 I can correctly assess clinical problems, solve practical nursing problems, provide health education, and respond to emergencies. |  |  |  |  |  |  |  |  |  |  |  |  |  |  |  |  |  | C55 When dealing with difficult clinical problems, I can remain calm and methodical, engage quickly, and summarize experience effectively. |

# Section C: Self-Assessment Table

For each indicator of the key risk factors for "Internet+ Nursing Services," how would you rate its performance?

| Level-1 indicator | Level-2 indicator | 1 | 2 | 3 | 4 | 5 | 6 | 7 | 8 | 9 | 10 |
| --- | --- | --- | --- | --- | --- | --- | --- | --- | --- | --- | --- |
| C1 Patient/Family-Related Risk Assessment | C11 The patient/family are polite and courteous to me. |  |  |  |  |  |  |  |  |  |  |
|  | C12 The patient/family have a relatively high educational level, good quality, and good self-cultivation. |  |  |  |  |  |  |  |  |  |  |
|  | C13 The patient/family have established a trusting relationship with me. |  |  |  |  |  |  |  |  |  |  |
|  | C14 The service effect is basically consistent with the expectations of the family/patient. |  |  |  |  |  |  |  |  |  |  |
| C2 External Environment Risk Assessment | C21 The weather on the service day is clear and suitable for travel, with no severe weather such as snow, fog, or strong wind. |  |  |  |  |  |  |  |  |  |  |
|  | C22 The distance between the service location and the departure point is appropriate. |  |  |  |  |  |  |  |  |  |  |
|  | C23 The transportation taken on the service day is safe. |  |  |  |  |  |  |  |  |  |  |
|  | C24 Road conditions are smooth on the service day and transportation is convenient. |  |  |  |  |  |  |  |  |  |  |
|  | C25 The community where the service location is situated is easy to find, and nearby transportation is convenient. |  |  |  |  |  |  |  |  |  |  |
|  | C26 The service community has sound security and a safe environment. |  |  |  |  |  |  |  |  |  |  |
|  | C27 The service recipient's home environment is safe, with no clutter blocking passage, overly slippery floors, or similar hazards. |  |  |  |  |  |  |  |  |  |  |
|  | C28 The service recipient's home and the nursing-operation environment have sufficient lighting. |  |  |  |  |  |  |  |  |  |  |
| C3 Platform/Hospital Supply Risk Assessment | C31 The Internet platform/hospital can provide effective supplies within their shelf life. |  |  |  |  |  |  |  |  |  |  |
|  | C32 The Internet platform/hospital can provide work-recording devices, such as video or photographic equipment, to keep the whole service process transparent. |  |  |  |  |  |  |  |  |  |  |
|  | C33 The Internet platform/hospital can provide a mobile-app location-tracking system and devices such as one-click alarms and delayed-warning devices to protect my personal safety. |  |  |  |  |  |  |  |  |  |  |
|  | C34 The Internet platform/hospital keeps information and data secure, and personal information is not easily disclosed. |  |  |  |  |  |  |  |  |  |  |
|  | C35 The Internet platform/hospital prices services reasonably. |  |  |  |  |  |  |  |  |  |  |
|  | C36 The Internet platform/hospital purchases medical liability insurance and personal accident insurance for nurses. |  |  |  |  |  |  |  |  |  |  |
| C4 Nurse Service-Related Experience | C41 My medical institution encourages nurses to provide home-based "Internet+ Nursing Services." |  |  |  |  |  |  |  |  |  |  |
|  | C42 I understand the entry qualifications for "Internet+ Nursing Services" nurses and the relevant laws and regulations. |  |  |  |  |  |  |  |  |  |  |
|  | C43 I have received systematic and comprehensive home-care training, including relevant "Internet+ Nursing Services" policies and regulations, management of difficult nursing problems, emergency nursing response, interpersonal communication, and professional competence. |  |  |  |  |  |  |  |  |  |  |
|  | C44 I have rich experience in "Internet+ Nursing Services" (participation in "Internet+ Nursing Services" ≥ 3 times). |  |  |  |  |  |  |  |  |  |  |
| C5 Assessment of Nurses' Clinical Core Competence | C51 My professional knowledge reserve closely matches the knowledge required for this service item. |  |  |  |  |  |  |  |  |  |  |
|  | C52 My nursing operation skills are highly proficient. |  |  |  |  |  |  |  |  |  |  |
|  | C53 I communicate well with patients and their families. |  |  |  |  |  |  |  |  |  |  |
|  | C54 I can correctly assess clinical problems, solve practical nursing problems, provide health education, and respond to emergencies. |  |  |  |  |  |  |  |  |  |  |
|  | C55 When dealing with difficult clinical problems, I can remain calm and methodical, engage quickly, and summarize experience effectively. |  |  |  |  |  |  |  |  |  |  |

*Performance score: 0 = very inconspicuous to 10 = very conspicuous; the columns above correspond to scores 1–10 for marking.*

# Section D: Basic Personal Information

## Nurse Information

1. Sex: A. Male	B. Female

2. Your age (years): A. ≤30	B. 31–40	C. 41–50	D. ≥50

3. Your highest educational attainment: A. Secondary vocational school	B. Junior college	C. Bachelor's degree	D. Master's degree or above

4. Your professional title: A. Nurse	B. Senior nurse	C. Supervising nurse	D. Associate chief nurse or above

5. Your years of work experience (years): A. ≤5	B. 6–10	C. 11–20	D. ≥21

6. Your department: ______________________________

7. Your years of work experience in home-care services: ______________________________

8. Your cumulative number of completed "Internet+ Nursing Services" orders (times): A. ≤5	B. 6–15	C. 16–30	D. ≥31

9. Your confidence in participating in "Internet+ Nursing Services": A. Very unconfident	B. Unconfident	C. Neutral	D. Confident	E. Very confident

10. Home-care service items you frequently provide (please select the top five):

| 1. Vital-sign measurement | 2. Physical cooling | 3. Oxygen inhalation | 4. Nebulized inhalation |
| --- | --- | --- | --- |
| 5. Blood glucose measurement | 6. Peripheral venous indwelling needle maintenance | 7. Intramuscular injection | 8. Subcutaneous injection |
| 9. Nasal feeding | 10. Nasogastric tube replacement | 11. Indwelling/replacement of urinary catheter | 12. Venous blood sampling |
| 13. Peripherally inserted central catheter (PICC) maintenance | 14. Stoma care | 15. Drainage tube maintenance | 16. Rehabilitation guidance |
| 17. Health assessment and guidance | 18. Other items |  |  |

If you select "18. Other items," please write the name of the home-care service item: ______________________________

*Explanation: For the respondent, this refers to how confident you are in the answers you provided; it may also be understood as your degree of confidence in your own answers to the whole questionnaire.*

This is the end of the questionnaire. Please rest assured that the information provided by respondents will be used only for academic research and will not be published separately or used for any other purpose. The researchers thank you again for your assistance.
